# Beyond Taxonomic Analysis of Microbiomes: A Functional Approach for Revisiting Microbiome Changes in Colorectal Cancer

**DOI:** 10.3389/fmicb.2019.03117

**Published:** 2020-01-23

**Authors:** Mohammad Hossein Norouzi-Beirami, Sayed-Amir Marashi, Ali Mohammad Banaei-Moghaddam, Kaveh Kavousi

**Affiliations:** ^1^Laboratory of Complex Biological Systems and Bioinformatics, Department of Bioinformatics, Institute of Biochemistry and Biophysics, University of Tehran, Tehran, Iran; ^2^Department of Biotechnology, College of Science, University of Tehran, Tehran, Iran; ^3^Laboratory of Genomics and Epigenomics, Department of Biochemistry, Institute of Biochemistry and Biophysics, University of Tehran, Tehran, Iran

**Keywords:** microbiota, taxonomic profiling, functional profiling, advanced colorectal adenoma, colorectal carcinoma, classification

## Abstract

Colorectal cancer (CRC) is one of the most prevalent cancers in the world, especially in developed countries. In different studies, the association between CRC and dysbiosis of gut microbiome has been reported. However, most of these works focus on the taxonomic variation of the microbiome, which presents little, if any, functional insight about the reason behind and/or consequences of microbiome dysbiosis. In this study, we used a previously reported metagenome dataset which is obtained by sequencing 156 microbiome samples of healthy individuals as the control group (Co), as well as microbiome samples of patients with advanced colorectal adenoma (Ad) and colorectal carcinoma (Ca). Features of the microbiome samples have been analyzed at the level of species, as well as four functional levels, i.e., gene, KEGG orthology (KO) group, Enzyme Commission (EC) number, and reaction. It was shown that, at each of these levels, certain features exist which show significant changing trends during cancer progression. In the next step, a list of these features were extracted, which were shown to be able to predict the category of Co, Ad, and Ca samples with an accuracy of >85%. When only one group of features (species, gene, KO group, EC number, reaction) was used, KO-related features were found to be the most successful features for classifying the three categories of samples. Notably, species-related features showed the least success in sample classification. Furthermore, by applying an independent test set, we showed that these performance trends are not limited to our original dataset. We determined the most important classification features at each of the four functional levels. We propose that these features can be considered as biomarkers of CRC progression. Finally, we show that the intra-diversity of each sample at the levels of bacterial species and genes is much more than those of the KO groups, EC numbers, and reactions of that sample. Therefore, we conclude that the microbiome diversity at the species level, or gene level, is not necessarily associated with the diversity at the functional level, which again indicates the importance of KO-, EC-, and reaction-based features in metagenome analysis. The source code of proposed method is freely available from https://www.bioinformatics.org/mamed.

## Introduction

Human microbiome consists of 10–100 trillion symbiotic microbial cells which are harbored by each person (Turnbaugh et al., 2007; Ursell et al., 2013; Magnúsdóttir et al., 2017), which in turn affects the human physiologic aspects such as metabolism, drug interactions, and a variety of diseases. Analysis metagenomic data are a popular approach to obtain insights on the host–microbiome interactions (Bermúdez-Humarán, 2016). Based on metagenomic sequences, it is estimated that about 500−1000 different bacterial species live in the human gut (Sommer and Bäckhed, 2013), which include approximately 3.3 million different bacterial genes. Some international consortia such as MetaHIT (Qin et al., 2010) and the Human Microbiome Project (HMP) (Huttenhower et al., 2012) have put intensive investments on microbiome research, which highlights the importance of the topic.

Gut microbiota plays a central role in the human digestive system, including digestion of food (Hooper et al., 2002), empowerment of the human immune system (Sommer and Bäckhed, 2013), protection of intestinal mucosa (Kaiko and Stappenbeck, 2014), and protection against pathogens (Lawley and Walker, 2013). For treating microbiome-related diseases, different methods have been proposed, including fecal microbiome transplantation (Bakken et al., 2011), prescribing probiotics (Gareau et al., 2010), and changing the diet in the form of probiotics to manipulate the microbiome (Cani et al., 2009).

With the increasing expansion of studies in the field of the human microbiome, advanced statistical and computational methods are used for analyzing such high-dimensional data, including the metagenomic sequences of gut microbiome (Waldron, 2018). Taxonomic profiling and functional profiling are the two major approaches that have been used for analyzing the microbiome data (Dhariwal et al., 2017).

Several different studies have been performed in the field of Taxonomic profiling on the association between colorectal cancer (CRC) and microbiome. In Feng et al. (2015), by using 156 samples, 10 bacteria with the most significant differential changes are recognized and introduced as the markers of CRC. In another study, two bacterial species, namely *Fusobacterium nucleatum* and *Peptostreptococcus stomatis*, were found to be over-represented in CRC samples (Yu et al., 2017). In another work, 18 bacterial genera have been found to have significant frequency changes in CRC compared to control samples (Alomair et al., 2018). Similar studies have been done on the CRC metagenomic sequences, resulting in different bacteria genera to be identified as CRC markers (Balamurugan et al., 2008; Sobhani et al., 2011; Wang et al., 2011; Chen et al., 2012; Ahn et al., 2013; Wu et al., 2013; Xu and Jiang, 2017; Mori et al., 2018) (see also Table 2). In another work on 156 healthy and CRC samples at different stages, 22 species were identified at the taxonomic level with a detection rate of AUC > 0.75 (Zeller et al., 2014).

In contrast to the taxonomic profiling approach, in some studies, functional variations in the genetic content of metagenomic samples are also taken into account. FishTaco, for example, uses an analytical and computational framework for integrating taxonomic and functional variations (Manor and Borenstein, 2017). Another functional study highlighted 20 microbial genes as the CRC biomarkers (Yu et al., 2017). In another similar work, over 130,000 genes have been found for which a significant difference was found between two of the three sample categories (control, advanced colorectal adenoma, and colorectal carcinoma) (Feng et al., 2015). In another study, metagenomic data obtained from 52 healthy and 52 CRC samples were analyzed. This study was performed at the taxonomic and functional level to identify several bacteria, genes, modules, and pathways in the samples which can be used for CRC detection. Furthermore, when an independent dataset was used for the same purpose, 39% of the markers, including genes, modules, and pathways, were identified again as important factors for CRC detection (Vogtmann et al., 2016).

In the present work, we present an alternative approach, which relies on the functional annotation of metagenomic sequences. In this approach, the contribution of genes, KEGG orthology (KO) groups, enzymes, and reactions are quantified, and consequently, used for detecting features which are useful for classifying different samples. Using this approach, we investigated how taxonomic changes are reflected in biochemical characteristics of microbiome at the functional level.

## Materials and Methods

### Metagenomic Colorectal Cancer Datasets

In this work, we used the metagenomic sequences reported in Feng et al. (2015). This dataset (in this study, we say “Dataset1”) includes 156 metagenomic shotgun-sequenced fecal samples, including 63 controls (Co), 47 advanced colorectal adenomas (Ad), and 46 colorectal carcinomas (Ca) samples. These samples had been sequenced using the Illumina platform and paired-end sequencing method (with average read length = 100 bp and insert size = 350 bp) (Feng et al., 2015). Personalized data, including lifestyle and diet, age, gender, BMI, blood pressure, blood glucose, and some other clinical information of the patients are also available (Feng et al., 2015). Also, “Dataset2” was used, as an independent data set, to evaluate the performance of the constructed models. This dataset (named originally “cohort1”) contains 80 samples of the human fecal metagenome, from 24 control, 27 colorectal adenoma, and 29 colorectal carcinoma individuals (Thomas et al., 2019). The number of paired reads of these samples is very different among the samples (min: ∼7 M paired reads, mean: ∼23.6 M paired reads, and max: ∼91 M paired reads). Therefore, we chose 60 samples of this data, such that 20 samples were selected for each of the control, adenoma, and carcinoma groups (min = ∼10.7 M, mean = ∼21.3 M, and max = ∼49.2 M). Details of the selected samples from Dataset2 are provided in Supplementary Table S10.

### Metagenome Analysis

To evaluate the data quality, we used FastQC v0.11.5. This software receives the file with the FASTQ format and presents its different properties including the number of reads, read lengths, GC content, and some different quality-control parameters.

To identify the bacterial taxa in each sample, we used MetaPhlAn v2.0 software (Truong et al., 2015). This software can determine the percentage frequency of the bacteria, viruses, and the archaea at different taxonomic levels. In the present study, both files of the paired-end sequences in each sample have been separately used for recognizing the present bacteria, and the results were averaged.

### The Reference Gene Catalog

In the original work, Feng et al. (2015) have used the assembly approach, following gene prediction, to determine the frequency of genes. In contrast, in the present study, read mapping to a reference gene catalog was used for obtaining the frequency of each gene in a metagenome. For this purpose, a previously reported human gut gene catalog, IGC, was used (Li et al., 2014). This gene catalog was obtained by combining the metagenomic sequences of 1018 previously reported samples from different European, American, and Chinese individuals, together with 249 newly sequenced metagenomic samples. In this catalog, 9.88 × 10^6^ genes, together with their corresponding proteins have been reported (Li et al., 2014). To construct this gene catalog, two previously existing gene catalogs [namely, the MetaHIT catalog (Qin et al., 2010), and the gene catalog reported by Human Metagenome Project (Consortium, 2012)] had been used. The metagenomic sequences had been used for producing this gene catalog by using the MOCAT pipeline (Kultima et al., 2012). Finally, they have been integrated by CD-HIT clustering algorithm by finding potential sequence redundancies (Li and Godzik, 2006).

### Mapping Reads to the Gene Catalog

MOSAIK mapping tool v2.2.3 was used for obtaining the frequency of the genes (Lee et al., 2014). This program maps the paired-end reads to a gene catalog by using hash tables for a chosen word length (which is equal to 15 in this work). In this mapping process, one of the following cases can occur: (i) both of reads are mapped to the same gene; (ii) a read is mapped to one gene but the other read is mapped to another gene; (iii) one read is mapped to one gene, while the other read is not mapped to any gene; (iv) neither of the two reads are mapped. In the first three cases, the frequency of the mappings is separately counted for every gene in the catalog. Mapping score is computed based on the default values of MOSAIK, with match score = 10, mismatch score = −9, gap opening penalty = −15, gap extension penalty = −1. A read is considered to be mapped to a gene when its mapping score was greater than or equal to zero.

### Mapping Genes to KEGG Database

After computing the frequency of each gene, one should assign each gene to a certain KO groups. For this purpose, we used GhostKOALA tool which belongs to the KEGG database (Kanehisa et al., 2016). GhostKOALA is an automatic server for metagenome sequence annotation. This tool obtains amino acid sequences as the input and, if possible, returns the KO identifier, i.e., a primary KO number and potentially, some secondary KO number. However, there might be several EC numbers for a KO group, or alternatively, one EC number for several KO groups.

By using this KO identifier, the Enzyme Commission (EC) numbers related to each KO group were obtained. In this stage, there might be no annotated enzyme in the KEGG for a certain KO group. Alternatively, their several enzymes might be associated with a certain KO group.

In the next step, using the EC numbers from the previous step, we get the reactions related to each EC number in the KEGG database. Each EC number might be associated with multiple reactions. In this study, we used only those reactions which are confirmed by IUBMB.

### Data Normalization

Before starting the normalization process, we removed those genes whose abundance was less than five mapped reads in all samples (Best et al., 2015). Then, we divided the number of reads mapped to each gene to the length of the gene, as we compute the “frequencies” of genes with different lengths to calculate KO, EC number, and reaction frequencies. Since, in most cases, the metagenomic data are sparse, we used the cumulative sum scaling (CSS) algorithm to compositional correction and normalization (Kumar et al., 2018). CSS corrects the bias detected in differential abundance data using total-sum normalization (TSS) (Paulson et al., 2013). The implementation of CSS algorithm in R (i.e., metagenomeseq1) has been shown to have a higher performance than similar algorithms on sparse data (Lee et al., 2017). To normalize our gene frequency data, we used the same implementation of CSS algorithm. Furthermore, this algorithm was applied to correct the compositional bias on taxonomic data at the genus and species level.

To obtain the normalized values of KOs, EC numbers, and reactions, we used normalized values of the genes in each sample. Suppose KO_*i*_ = {*g*_1_, *g*_2_, …, *g*_*k*_} includes *k* genes, which means that genes *g*_1_, *g*_2_, …, *g*_*k*_ are associated with KO*_*i*_*. In this case, the normalized frequencies of these genes are summed in each sample to compute the KO*_*i*_* frequency. We perform the same procedure to compute the frequencies of EC numbers and reactions.

### Feature Selection

After computing the normalized frequencies of genes, KO groups, EC numbers, and reactions in each sample, one may exploit these features to classify samples to different categories. Two strategies can be considered for feature selection. The first strategy is unsupervised feature selection. In this strategy, feature selection is done regardless of data labels and only by considering the separating power of features (Du and Shen, 2015). The second strategy is supervised feature selection, where features are selected based on the data labels such that the samples are classified in the best way (Chandrashekar and Sahin, 2018). In the present work, the data labels are known. Therefore, it is possible to use these labels in the feature selection process.

For selecting features from the existing sets (namely gene, KO groups, EC numbers, and reaction), we used the multi-cluster feature selection (MCFS) algorithm (Cai et al., 2010). MCSF selects a subset of features so that the selected features preserve the multi-cluster characteristic of the data. This algorithm, by default, works as a supervised feature selection method. In the present work, supervised mode of the MCFS is used.

### Statistical Analyses

The non-parametric Kruskal–Wallis *H* test was used to determine whether two or more samples that follow the same distribution. This test can be considered as a generalization of Mann–Whitney *U*-test, which can be used merely for comparing two groups of samples. The parametric equivalent of this test is one-way analysis of variance (ANOVA). To correct for the false discovery rate we used the Benjamini–Hochberg (BH) method (Corder and Foreman, 2009).

For pairwise comparison of the normalized reaction frequencies in different categories of cancer progression (i.e., Co vs. Ad and Ad vs. Ca) we used the Mann–Whitney *U*-test. Based on these pairwise comparison results, we categorize each reaction to one of the nine possible trend schemes (Table 5).

### Cross-Validation and Classification Based on Support Vector Machine

The selected features (see above) were used to train a support vector machine (SVM) to classify the samples. SVM is a classification technique that was first introduced by Cortes and Vapnik (Vapnik, 1995). SVM was initially designed for binary classification but was later generalized for multi-class modes. This technique tries to minimize the classification error by maximizing the distance between the hyperplanes and data points (Borah and Gupta, 2017). To evaluate the performance of trained SVM, 100 repeats of 10-fold cross-validation were used.

## Results

In the present work, we investigated how the functions encoded by gut microbiome may change during CRC progression. We used two previously published datasets. “Dataset1” consists of 156 samples extracted from feces of 63 healthy (Co), 47 advanced colorectal adenomas (Ad), and 46 colorectal carcinomas (Ca) individuals (Feng et al., 2015). This dataset is used to extract features as well as construct prediction models. “Dataset2” was used, as an independent data set, to evaluate the performance of the constructed models. This dataset contains 80 samples of the human fecal metagenome, from 24 control, 27 colorectal adenoma, and 29 colorectal carcinoma individuals (Thomas et al., 2019). Then, each gene was analyzed to determine its related KO group(s), EC number(s), and reaction(s). Furthermore, the normalized metagenomic frequency of each gene, KO group, EC number, and reaction was calculated.

### Data Quality Control

We used FastQC software to control the quality of the dataset. This dataset had been already preprocessed and no special qualitative problem was found. Table 1 summarizes the quality information of the 156 samples. Further details about the characteristics of the reads in this dataset are presented in Supplementary Table S1.

**TABLE 1:** The statistical summary of the metagenomic sequences used in the present work.

Total number of paired reads	4,109,778,973
Average number of paired reads in each sample	26,344,737
Average read length	91.3 bp
Average GC content	46.4%

### Identification of Bacterial Taxa in the Samples

**FIGURE 1:**
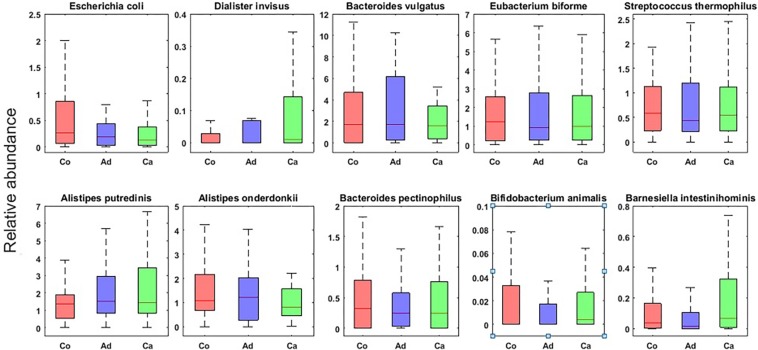
Top 10 bacterial species (with the highest normalized frequency values) in control (Co), adenoma (Ad), and carcinoma (Ca) groups with *p*-value ≤ 0.05 in Kruskal–Wallis test.

In this research, using MetaPhlAn, we detected the bacteria at the genus and species level. The detailed taxonomic profiles of all samples are presented in Supplementary Table S2. In the next step, by using Kruskal–Wallis statistical test, we found those bacteria whose frequencies are changed significantly among the groups (*p*-value ≤ 0.05) over the Co, Ad, and Ca categories at the genus and species level. Table 2 summarizes the list of those bacterial genera which are reported as the markers of CRC in other metagenomic studies. Additionally, Figure 1 shows the top 10 species, based on the MetaPhlAn2 analysis, in the three categories of Co, Ad, and Ca.

**TABLE 2:** The bacterial genera with a significant difference in control and CRC.

	Present study	Alomair et al., 2018	Xu and Jiang, 2017	Mori et al., 2018	Balamurugan et al., 2008	Sobhani et al., 2011	Chen et al., 2012	Wang et al., 2011	Ahn et al., 2013	Wu et al., 2013
*Ruminococcus*									✓	
*Bifidobacterium*	✓									
*Eubacterium*				✓			✓			
*Bacteroides*	✓		✓			✓		✓		✓
*Blautia*				✓						
*Faecalibacterium*					✓					
*Escherichia*	✓		✓	✓	✓			✓		
*Collinsella*		✓					✓			
*Dorea*				✓						
*Klebsiella*	✓									
*Alistipes*	✓				✓					
*Streptococcus*	✓		✓					✓		
*Roseburia*								✓		✓
*Clostridium*	✓									
*Prevotella*	✓					✓				
*Parabacteroides*	✓									
*Anaerostipes*				✓						
*Dialister*	✓									
*Barnesiella*	✓									
*Enterococcus*								✓		
*Oscillibacter*	✓									
*Bilophila*	✓				✓					
*Megasphaera*	✓									
*Porphyromonas*	✓	✓					✓	✓	✓	
*Odoribacter*	✓									
*Paraprevotella*	✓						✓			
*Fusobacterium*	✓	✓	✓						✓	✓
*Acidaminococcus*	✓									
*Slackia*							✓			
*Anaerotruncus*	✓						✓			
*Peptostreptococcus*	✓	✓					✓	✓		
*Sutterella*	✓			✓						
*Shigella*	✓							✓		
*Gemella*	✓	✓								
*Desulfovibrio*	✓						✓			
*Parvimonas*	✓				✓					
*Solobacterium*		✓								
*Atopobium*		✓							✓	
*Burkholderia*		✓								
*Anaerococcus*							✓			
*Peptoniphilus*		✓								
*Finegoldia*		✓								
*Selenomonas*		✓								
*Comamonas*		✓								
*Yersinia*		✓								
*Listeria*		✓								

*The second column represents the data obtained by MetaPhlAn (in the present study).*

### Mapping Reads to Genes, and Genes to KO Groups, EC Numbers, and Reactions

By using MOSAIK, we mapped the (paired-end) reads onto the IGC gene catalog. In general, 84% of the total reads were mapped to the IGC. More specifically, 64% of the read pairs have been both mapped to the same gene, while 27% of read pairs were mapped to separate genes, or, one read has been mapped to a gene and the other read has not been mapped to any gene. The remaining 9% of paired reads have not been mapped to any gene. Finally, we found 5,697,860 genes in the IGC catalog to which at least one read was mapped. The number of the mapped reads of each sample is presented in Supplementary Table S3.

We used the GhostKOALA tool to obtain the KO groups associated with each gene sequence. At the end, from among the 5,697,860 genes to which at least one read has been mapped, for 1,937,738 genes the primary KO group (and also, for 4,111,878 genes at least one KO group) were obtained (Supplementary Data Sheets S1, S2, respectively). Overall, 19,001 unique KO numbers have been found for all the genes. For these KO numbers, 6,349 KO numbers have an annotated EC number (Supplementary Table S4). At this stage, we reached 3,846 unique EC numbers in KEGG, which corresponds to 1,869,711 genes (Supplementary Data Sheet S3). From these 3,864 EC numbers, 3,224 EC numbers have annotated reactions (Supplementary Table S5). However, there are 3,702 annotated reactions, which correspond to 1,476,691 genes (Supplementary Data Sheet S4).

### The Altered Frequency of Genes Is Less Pronounced Compared to Their Assigned KO Group, EC Number, and Reactions in Metagenome Data of Group Samples

The Kruskal–Wallis was applied to test the significance of differences in the normalized frequencies of the gene, KO groups, EC number, and reaction obtained for each sample. The normalized frequencies of each gene in each of the samples are presented in Supplementary Data Sheet S6. Other normalized frequencies of KO groups, EC numbers, and reactions are calculated from normalized gene frequencies and are presented in Supplementary Tables S6–S8.

In each case of the Kruskal–Wallis test, the statistical variable is considered as a single species, a gene, a KO group, an EC number, or a reaction. For each of the mentioned variables, there are 156 samples which belong to the three categories of Co, Ad, and Ca, and the number of the samples in each group is, respectively, 63, 47, and 46. For example, according to the results (Table 3 and Supplementary Table S9), out of 4,706,619 analyzed genes, 612,356 genes showed a significant change (*p*-value ≤ 0.05). To correct for the false discovery rate we used the BH method. After *p*-value adjustment, 2.1% of 4,706,619 genes were significant at Kruskal–Wallis test (*p*-value ≤ 0.05).

**TABLE 3:** The results of the Kruskal–Wallis test for significance level (*p*-value ≤ 0.05) at different levels (species, gene, KO group, EC number, and reaction).

Test level	Total sample	Number of significant cases (*p*-value ≤ 0.05)	Percentage of significantly changed cases	Percentage of significantly changed cases after *p*-value adjustment
Species	496	87	17.54	5.04
Gene	4,706,619	612,356	13.01	2.10
KO group	19,001	2,492	13.11	0.44
EC number	3,864	422	10.92	0.50
Reaction	3,702	409	11.05	0.52

### Determining Over-Represented Genes, KO Groups, EC Numbers, and Reactions

In this step, we identified whether each gene (or, their assigned KO group, EC number, or reaction) with a statistically significant altered frequency is specifically over-represented in each category of samples, i.e., Co, Ad, and Ca. A gene is considered to be “present” in a metagenome sample if (i) at least one read is mapped to it, and (ii) the frequency of its mapped reads is among the 90% of the top-ranking frequencies of the reads mapped to the other genes of that sample. To claim if a gene is specific to a sample group, a threshold of *b* was defined. For example, when *b* = 0.95 it means that a gene is assumed to be “special” in that sample group if it is present in the metagenome of 95% of individuals of that sample group. The same criteria were applied for determining the presence and special of KO groups, EC numbers, and reaction in each sample group.

**FIGURE 2:**
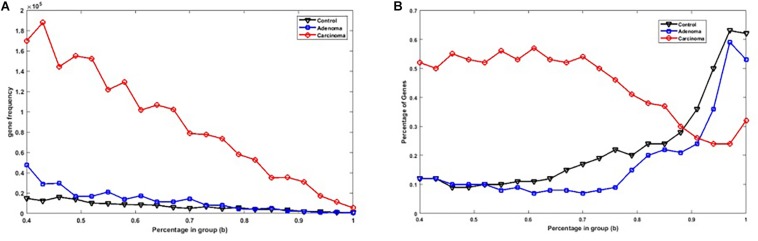
**(A)** The number of specific genes in each sample group with different *b-*values. **(B)** The percentage of specific genes in each sample group with significantly altered frequencies at the level of *p*-value ≤ 0.05 according to the Kruskal–Wallis test.

**FIGURE 3:**
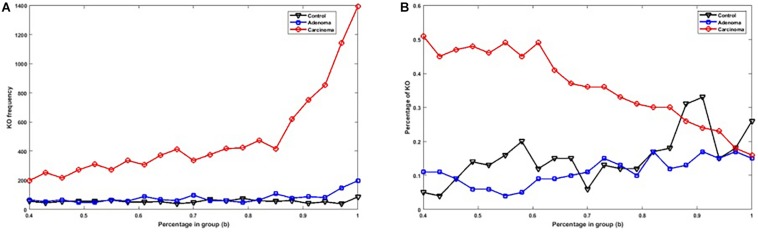
**(A)** The number of specific KO groups in each sample group with different *b-*values. **(B)** The percentage of specific KO groups in each sample group with significantly altered frequencies at the level of *p*-value ≤ 0.05 according to the Kruskal–Wallis test.

**FIGURE 4:**
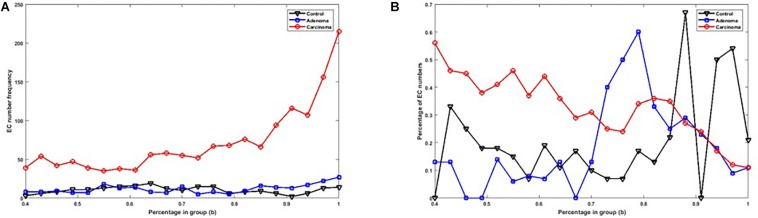
**(A)** The number of specific EC numbers in each sample group with different *b-*values. **(B)** The percentage of specific enzymes in each sample group with significantly altered frequencies at the level of *p*-value ≤ 0.05 according to the Kruskal–Wallis test.

**FIGURE 5:**
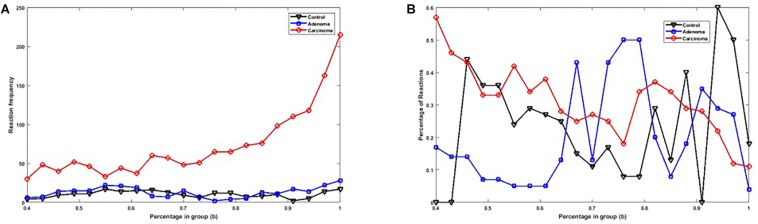
**(A)** The number of specific reactions in each sample group with different *b-*values. **(B)** The percentage of specific reactions in each sample group with significantly altered frequencies at the level of *p*-value ≤ 0.05 according to the Kruskal–Wallis test.

Figure 2A shows the number of specific genes in each sample category, for different *b*-values (i.e., if a gene is solely present in one of the three categories and is not special in any of the other categories). Figure 2B shows the percentage of present genes (as shown in Figure 2) whose frequencies are significantly changed over sample categories (*p* ≤ 0.05 in Kruskal–Wallis test). Similarly, the number of present KO groups, EC numbers, and reactions of the three categories are represented in Figures 3A, 4A, 5A, respectively. The percentage of significantly changed specific KOs, enzymes, and reactions in each sample group is represented in Figures 3B, 4B, 5B, respectively.

Note that based on Figures 2A, 3A, 4A, 5A, when *b* is increasing, the number of special genes is generally decreasing, while the number of special KO groups, EC numbers, and reactions in that range generally show an ascending trend.

**FIGURE 6:**
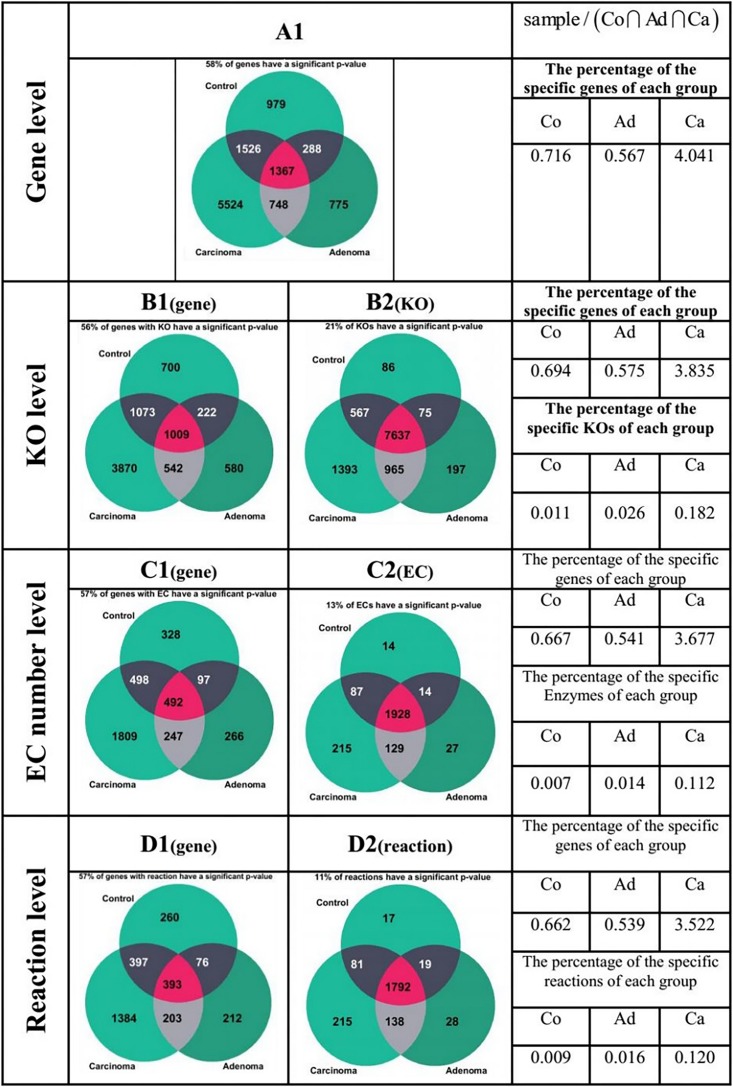
Comparison of specificity of the genes in different forms of the gene, KO, enzyme, and reaction. The green area in each of the Venn diagrams represents the special section for each of the sample groups, while the purple region is the common area among all the sample groups.

Figure 6 presents a different state of the specific genes for the different sample group of Co, Ad, and Ca. All the Venn diagrams of Figure 6 are drawn for *b* = 1 (meaning that the given specific gene, KO group, EC number, or reaction is present in all individual of the corresponding sample category). The Venn diagram in Figure 6(A1) illustrates the 4,706,619 genes to which at least five reads have been mapped at all samples. Out of 7278 present genes (sum of 5524, 775, and 979 gene), there are 58% of genes whose frequencies were significantly changed among different groups (*p* ≤ 0.05 in Kruskal–Wallis test). On the other hand, the values at the right side of Figure 6 have been obtained by dividing the number of the present genes of each sample group (green area) by the number of genes which are present in the intersection (purple area). For example, the result of dividing the number of present genes in Co by the number of genes in the intersection is 0.79. In Figure 6(B2), Venn diagram has been drawn for the 19,001 KOs obtained from KEGG database. Here, only 21% of the present KO groups of each category have a *p* ≤ 0.05 according to the Kruskal–Wallis test. For example, the ratio of control-specific KO groups (green area) to shared KO groups (purple area) is only 0.011, which is considerably smaller compared with the value of present genes in Figure 6(A1). As shown in Figure 6(B1) Venn diagram, 4,111,878 genes in KEGG have assigned annotated KO. Consequently, one may argue that the specific genes of Figure 6(A1) may be missed in Figure 6(B2) (due to lack of KO annotation). By comparing the percentages related to the genes in Figure 6(A1) and (B1), no egregious difference is observed. The ratio for present EC numbers in Figure 6(C2) is even lower. This diagram has been drawn for the 3864 enzymes which have an annotation in KEGG. Among the specific EC numbers of each sample category, only 13% have a *p* ≤ 0.05 (Kruskal–Wallis test). However, Venn diagram of Figure 6(C1) has been drawn for the 1,869,711 genes which have an annotated EC number in KEGG. By comparing the percentages related to the genes in Figure 6(C1), one again observes that there is no considerable difference between the Venn diagram related to the genes in the two previous section. Finally, in Figure 6(D2) Venn diagram has been drawn for the 3702 reactions annotated in KEGG and among the specific reactions of each group, only 11% of them have a *p*-value ≤ 0.05 in Kruskal–Wallis test. The ratio of the specific reaction of each group to the joint section is lower compared to the corresponding value for the gene. However, Venn diagram of Figure 6(D1) has been drawn for the 1476,691 genes which have an annotated reaction in KEGG. The pattern observed in this diagram does not have a significant difference with the gene diagrams in the previous sections, either.

### Identity of Changed Reactions in Different Categories

To determine the functional changes at the level of reactions in the metagenome of individuals among Co, Ad, and Ca groups, we compared the normalized frequencies of reactions between Co and Ad, and also between Ad and Ca categories. For this purpose, we used the Mann–Whitney *U*-test with the significance level of *p* ≤ 0.05. In this case, nine cases can occur (Table 4). The results of this statistical test are presented in Supplementary Table S11 in detail. Interestingly, there is only one reaction that shows a monotonically decreasing frequency trend, while there is another reaction that shows a monotonically increasing frequency trend.

**TABLE 4:** The nine conditions obtained for the reactions by using the Mann–Whitney *U*-test.

State of changing abundance of reaction between three groups	Number of reactions
(1) Co = Ad and Ad = Ca	3267
(2) Co = Ad and Ad < Ca	140
(3) Co = Ad and Ad > Ca	184
(4) Co < Ad and Ad = Ca	75
(5) Co < Ad and Ad < Ca	1*
(6) Co < Ad and Ad > Ca	14
(7) Co > Ad and Ad = Ca	15
(8) Co > Ad and Ad < Ca	6
(9) Co > Ad and Ad > Ca	0

For example, in the second row, “Co = Ad and Ad < Ca” means that the abundance of 140 reactions in this state is constant from control to adenoma and increased from adenoma to carcinoma. *The only reaction here is: L-glutamate + L-aspartate 4-semialdehyde <=> 2-oxoglutarate + L-2,4-diaminobutanoate.

### Classification Based on Selected Marker Features

As explained above, we obtained five normalized datasets, i.e., bacterial species, gene, KO group, EC number, and reaction. Now, by using this data, we try to select those features which accurately classify the samples into three Co, Ad, and Ca categories.

We use the MCFS algorithm to select the features (Cai et al., 2010). Table 5 presents the optimum number of features selected for the five datasets in supervised manners.

**TABLE 5:** The number of the features selected by the MCFS algorithm.

	Number of features in the supervised method
Bacterial species	64
Gene	1387
KO group	130
EC number	106
Reaction	124

The selected features in Table 6 were used to classify samples by SVM. Next, we used 100 repeats of 10-fold cross-validation for execution of SVM classifier. Tables 6, 7 represent the values of accuracy, specificity, sensitivity, and the area under the ROC curve (AUC) of classification. The AUC measure was calculated using the Wilcoxon rank sum test. Table 6 shows the results of SVM, for which, both the training and the test sets were selected from Dataset1. In contrast, Table 7 shows the results of SVM when the training set is obtained from Dataset1, while the test set is selected from Dataset2.

**TABLE 6:** The average results of the 100 repeats of 10-fold cross-validation and execution of SVM for selected features.

	SVM with selected features on first dataset				
	Number of features	Accuracy	Sensitivity	Specificity	AUC
Species	64	0.752	0.579	0.818	0.741
Gene	1387	0.788	0.677	0.841	0.782
KO group	130	0.854	0.762	0.899	0.849
EC number	106	0.812	0.705	0.862	0.814
Reaction	124	0.773	0.645	0.832	0.783

*The training and test data are selected from the Dataset1.*

**TABLE 7:** The results of SVM classification for independent dataset.

	SVM trained with first dataset and tested with second dataset				
	Number of features	Accuracy	Sensitivity	Specificity	AUC
Species	64	0.641	0.446	0.738	0.593
Gene	1387	0.652	0.464	0.747	0.592
KO group	130	0.733	0.600	0.800	0.706
EC number	106	0.680	0.510	0.765	0.668
Reaction	124	0.653	0.455	0.753	0.597

*The training data are selected from the Dataset1 and the test data from the second Dataset2.*

According to the results presented in Table 6, KO-based features are better than species-, gene-, EC-, and reaction-based features at predicting the sample categories. Table 6 suggests that classification by genus- or species-based features, which have been used for classification of samples in previous studies (classification accuracy of 75.2% and AUC of 74.1%; see Table 2) has the lowest accuracy among the features presented in Table 6. Here, we reached the classification accuracy of 85.4% and AUC of 84.9% in KO-based features.

Tables 6, 7 indicate that classification by species- and gene-based features has the lowest accuracy among the studied features. Gene-based features have been the basis of decision making in some previous studies (Yu et al., 2017). Our results suggest that the difference in relative abundance at the species level and gene count does not have the same group-associated differences in Co, Ad, and Ca samples.

We also use Dataset2, as an independent dataset, to evaluate the performance of the model constructed using Dataset1. This dataset contains 80 healthy, adenoma, and carcinoma samples but we use 60 samples of Dataset2 (see the section “Materials and Methods”). We then used the same data processing procedure that was previously applied on Dataset1 to compute the normalized frequency of different features in Dataset2.

Finally, the same features as those selected for the Dataset1 in Table 5 are extracted from Dataset2. The trained SVMs using the features extracted from Dataset1 are then evaluated with the features extracted from Dataset2. Table 7 shows the results of this evaluation. The results in Table 7 also confirm that KO-based features have the highest classification power among the five specified feature categories.

Tables 6, 7 indicate that classification by species- and gene-based features has the lowest accuracies among the selected features. Gene-based features have been the basis of decision making in the previous study (Yu et al., 2017). Our results suggest that the change in the frequencies of the bacterial species and genes do not have the same association with functional changes of the microbiome.

### Comparison of Over-Represented Genes With Previously Reported CRC Biomarkers

We compared the list of genes obtained in this research with the list of previously reported CRC biomarkers (Yu et al., 2017). In that study, 20 genes have been reported as CRC markers. Furthermore, among these CRC markers, four genes had been confirmed by the results of an independent work (Yu et al., 2017). The sequence data of these 20 biomarker genes are presented in Supplementary Data Sheet S5. We blasted these 20 genes to the dataset of genes in Table 6 to find if there is any correspondence between these two gene sets. The results of this analysis are presented in Supplementary Table S12. Overall, 13 out of 20 genes were found to be present among the results of our study. More specifically, all of the four genes which were reported to be confirmed in Yu et al. (2017) were among the genes that were selected in our work.

## Discussion

In this research, we reanalyzed metagenomic sequences of 156 control, advanced colorectal adenoma, and colorectal carcinoma samples (Feng et al., 2015). The sequence reads were mapped to a catalog of 9.88 million genes (Li et al., 2014). Then, by using the comprehensive annotation information of the KEGG database, we extracted further functional features at the levels of KO groups, EC numbers, and reactions. From these functional features, we selected those features which could separate Co, Ad, and Ca samples with an accuracy of 85.4% and AUC of 84.9%. However, our results suggest that the accuracy of classification by using species- and gene-based features is far less than those of KO-, EC-, and reaction-based features. From these results, one can conclude that the differences between the frequency of bacteria and genes are not fully reflected in the differences at the functional levels. In other words, taxonomic and genetic changes in metagenome are not necessarily associated with functional changes. Similar conclusions have been mentioned previously in the literature (Flores et al., 2014; Tian et al., 2017). In the present study, we used a computational quantitative approach to identify the relative importance of features at various functional levels, including gene, KO, EC, and reaction.

Also, according to Figures 2–5, when *b* is closer to one (meaning that the components are specified in all sample groups), a descending trend is observed for the gene level. However, at the level of functional features, i.e., KO group, EC number, and reaction, the trends are ascending.

Another important observation of this work is that the ratio of carcinoma-specific genes to the common genes in Figure 6 is higher compared to that ratio for adenoma-specific and control-specific samples. This observation suggests that the microbiome dysbiosis in colorectal carcinoma results in an increased gene diversity among the carcinoma samples. Related observations have been previously reported in the literature (Burns et al., 2015), but the precise role of the increase in bacterial diversity in CRC is yet to be understood.

In the present work, we used the IGC gene catalog (Li et al., 2014) as a comprehensive list of genes reported to be present in the human gut microbiome. In any gene catalog, including the one used in the present work, two or more different genes may be in the same KO group. As a result, grouping genes of the same KO group can increase the accuracy of decision-making about the healthy and patient samples. This is presumably the reason behind the success of KO-based features in classification.

In this study, from the 156 samples, we obtained about 5.7 million genes with non-zero frequency, while for 4.1 million genes at least one KO number is specified. Furthermore, for 1.9 million genes, at least an EC number is found in KEGG. This value decreases to 1.5 million genes with annotated reactions. Therefore, one may argue that when we use KO groups, EC numbers, or reactions for classification, some of the KO groups, EC numbers, or reactions may be discarded, simply due to missing annotations in KEGG. However, according to Figure 6, it can be concluded that in case of the genes which show a significant difference, the significance level decreases by moving from KO group to EC number, and the reaction. Note that the data of KO-based features alone have the highest importance and can detect the healthy and patient samples with an accuracy of 85.4%. A possible explanation for the highest classification accuracy of KO is that for certain KO groups, there is no annotated EC numbers or reactions.

We also classified the samples of Dataset2 using the features selected from Dataset1. When the predictive power is evaluated, we observed that although classification accuracy for Dataset2 is lower than that of Dataset1, the same trend is observed again, as the best performance is obtained by applying KO-based features (accuracy = 73.3% and AUC = 70.6%).

In this study, we found 26 bacterial genera and 10 bacterial species which showed significant differences among the control, advanced adenoma, and carcinoma groups. Some of these bacteria have been reported as CRC biomarkers in previous studies (Table 2).

In this research, we calculated the changes in the frequency of all the gut microbiome-related reactions which have been annotated in KEGG from the healthy state to the adenoma, and from adenoma to the carcinoma state. Also by using statistical test, we have determined that which of the 3702 annotated reactions have had significant frequency changes. In general, we have specified nine states for these reactions and all of these reactions are presented in Supplementary Table S11 in detail. For example, the only reaction which always shows an increasing trend (shown by ^∗^ in Table 4) is R06977. In this reaction, aspartate and glutamate amino acids are consumed. This reaction shows an increasing trend from healthy to adenoma states, and also, from adenoma to carcinoma states. In previous works, increased level of aspartate and glutamate amino acids in CRC has been reported (Okada et al., 1993). On the other hand, it has been reported that the levels of these two amino acids are correlated to the growth of the colorectal tumors (Yoshioka et al., 1992). Therefore, one can conclude that microbiome activities, and especially its glutamate metabolism, play an important role because of its strong correlation with the metabolism of colorectal cancer cells.

In the same context and as other representative, two other reactions used as feature were R01080 and R02353 (rows 2 and 8 of Table 4, respectively), which showed significant increment in their frequency in colorectal carcinoma. R01080 is a reaction for hydrolysis of uridine to uracil and belongs to the pyrimidine metabolism pathway. It has been shown that the recycling of uracil is vital for the growth of strains with inability of *de novo* pyrimidine synthesis (Kurtz et al., 2002). On the other hand, nucleotide metabolism is of great importance in cancer. For example, capecitabine, which is a fluoropyrimidine drug, is used in chemotherapy of several cancers including CRC and act by inhibition of nucleoside metabolism, and consequently, cell division. Our findings suggest that the microbiome of a patient in the advanced CRC stage may pave the way for the progression of cancer by providing tumor with further uracil, which is necessary for cell growth (Walko and Lindley, 2005). Reaction R02353 represents the conversion of 17beta estradiol to the estrone, which is catalyzed by 7 beta-hydroxysteroid dehydrogenase (17HSD). It has been shown that there is an association between the activity of this enzyme and cell proliferation, and additionally, migration ability of breast cancer cells (Vihko et al., 2005; Aka et al., 2012, 2017). On the other hand, R00618 (Row 7 of Table 2) shows a decrease in adenoma and carcinoma samples compared to the normal ones. This reaction is catalyzed by thiamine triphosphatase, which convert thiamin triphosphate to the thiamin diphosphate (active form of Vitamin B1). Interestingly, it has previously been shown that high doses of Vitamin B1 may reduce the proliferation of cancer cells (Hanberry et al., 2014). The observed decrements of this reaction in patients with adenoma and carcinoma suggest that in these patients microbiome functionality is changed in a way that produce less active form of Vitamin B1, which means that cancer cell proliferation is not inhibited.

## Conclusion

In the present study, our goal was to understand, at the functional level, how microbiome changes are associated with diseased states in CRC. Using a rigorous secondary data analysis approach (Longo and Drazen, 2016), we tried to find the relevant taxonomic and functional features that can predict the disease. We used the MOSAIK tool to map the metagenomic reads to the comprehensive IGC gene catalog. Then, for each gene, its associated bacterial species, as well as its KO group, EC number, and reaction were extracted when possible. We showed that species- or gene-based features alone are not especially good at classifying control and the disease samples, compared to KO-, EC-, and reaction-based features, which are suggested to be used in the present study. Moreover, using a machine learning method, we showed that it is possible to achieve >85% prediction accuracy.

## Data Availability Statement

Publicly available datasets were analyzed in this study. This data can be found here: https://www.ebi.ac.uk/ena/data/view/PRJEB7774 and https://www.ebi.ac.uk/ena/data/view/PRJEB27928.

## Ethics Statement

The current study does not include any sampling from patients. A total of 156 metagenomic samples used in this study has been obtained from another study that was approved by the local ethics committee (Ethikkommission des Landes Salzburg, approval no. 415-E/1262/2-2010) and informed consent was obtained from all participants.

## Author Contributions

MN-B performed the data preprocessing and analysis, constructed the models, and drafted the manuscript. KK and S-AM contributed to the initial idea. MN-B and KK designed the computational aspects of work. S-AM and AB-M contributed to the biological insights. KK, S-AM, and AB-M critically revised the manuscript. All authors contributed to visualize the results and to the discussion, and gave the final approval for publication.

## Conflict of Interest

The authors declare that the research was conducted in the absence of any commercial or financial relationships that could be construed as a potential conflict of interest.

## Supplementary Material

TABLE S1 Statistical information about the characteristics of the reads in samples obtained using FastQC software.

TABLE S2 The detailed taxonomic profiles of all samples obtained using MetaPhlAn.

TABLE S3 Statistical information on the number of reads in samples mapped to the gene catalog using the MOSAIK tool.

TABLE S4 The annotated EC number associated with each KO obtained from the KEGG database.

TABLE S5 The annotated reaction associated with each EC number obtained from the KEGG database.

TABLE S6 The normalized frequencies of each KO in each of the 156 samples of the dataset. In this data, the rows indicate the samples and the columns indicate the frequency of each KO in the sample.

TABLE S7 The normalized frequencies of each EC number in each of the 156 samples of the dataset. In this data, the rows indicate the samples and the columns indicate the frequency of each EC number in the sample.

TABLE S8 The normalized frequencies of each reaction in each of the 156 samples of the dataset. In this data, the rows indicate the samples and the columns indicate the frequency of each reaction in the sample.

TABLE S9 The results of the statistical Kruskal–Wallis test are based on the normalized frequencies of the gene, KO, EC number, and the reaction, which sorted with the *p*-value.

TABLE S10 Total information about Dataset2.

TABLE S11 This dataset shows the information related to the state of each reaction from healthy to adenoma and from adenoma to carcinoma, which is obtained using the Mann–Whitney *U*-test with *p*-value < 0.05. The total reactions are divided into nine groups and show the characteristics of the reactions in each group.

TABLE S12 The results of the blast of the nucleotide sequence of the genes obtained in Table 6 on the genes identified as markers in Yu et al. (2017).

DATA SHEET S1 The primary KO is mapped to each gene sequence using the GhostKOALA tool.

DATA SHEET S2 The total KO (primary and secondary) is mapped to each gene sequence using the GhostKOALA tool.

DATA SHEET S3 The annotated EC number associated with each gene sequence obtained from the KEGG database.

DATA SHEET S4 The annotated reaction associated with each gene sequence obtained from the KEGG database.

DATA SHEET S5 The nucleotide sequence of the 20 genes referred to as a marker for colorectal cancer is presented in Yu et al. (2017).

DATA SHEET S6 The normalized frequencies of each gene in each of the 156 samples of the dataset. In this data, the rows indicate the samples and the columns indicate the frequency of each gene in the sample.
